# Expression Characteristics and Prognostic Value of KLRG2 in Endometrial Cancer: A Comprehensive Analysis Based on Multi-Omics Data

**DOI:** 10.3390/biomedicines13071592

**Published:** 2025-06-30

**Authors:** Xiaoyan Huang, Ailian Li, Dianbo Xu

**Affiliations:** Department of Gynecology, the Affiliated Jiangning Hospital of Nanjing Medical University, Nanjing 211112, China

**Keywords:** endometrial neoplasms, KLRG2, biomarkers, tumor microenvironment, prognosis, methylation

## Abstract

**Background**: Endometrial cancer (EC) remains a major gynecologic malignancy with limited biomarkers for risk stratification. While killer cell lectin-like receptor G2 (KLRG2) exhibits oncogenic properties in other cancers, its clinical significance and mechanistic roles in EC are unknown. This study aims to systematically characterize KLRG2 expression in EC, evaluate its prognostic significance, decipher underlying molecular mechanisms, and explore its role in tumor immune microenvironment regulation. **Methods**: We performed integrated multi-omics analyses using TCGA-UCEC (n = 552), GTEx, and GEO cohorts (GSE106191), complemented by qPCR validation (14 EC vs. 14 normal samples). Prognostic models were constructed via Cox regression and time-dependent ROC analysis. Epigenetic regulation was assessed through methylation profiling (UALCAN/MethSurv), and immune correlations were evaluated using TIMER/ESTIMATE algorithms. **Results**: KLRG2 was significantly overexpressed in EC tissues compared to normal endometrium (*p* < 0.001), validated by immunohistochemistry and qPCR. High KLRG2 expression independently predicted worse overall survival (HR = 3.08, 95% CI = 1.92–4.96) and progression-free interval (HR = 1.98, 95% CI = 1.37–2.87). Furthermore, elevated KLRG2 levels were significantly associated with advanced-stage disease (*p* < 0.001), deep myometrial invasion (*p* < 0.05), and high-grade histology (*p* < 0.001). Mechanistically, promoter hypomethylation was associated with KLRG2 overexpression (*p* < 0.001), while hypermethylation at three CpG sites (cg04915254, cg04520485, cg23104233) correlated with poor prognosis. Functional enrichment linked KLRG2 to cell cycle checkpoints and G Protein-Coupled Receptor signaling. Immune profiling revealed cytotoxic lymphocyte depletion (CD8+ T cells: Spearman’s ρ = −0.247, *p* < 0.001; NK CD56bright cells: Spearman’s ρ = −0.276, *p* < 0.001) and Th2 polarization (Spearman’s ρ = 0.117, *p* = 0.006). **Conclusions**: This comprehensive EC study establishes KLRG2 as a dual diagnostic/prognostic biomarker and immunomodulatory target. These findings provide a rationale for developing KLRG2-directed therapies to counteract tumor-intrinsic proliferation and microenvironmental immune suppression. Future single-cell analyses are warranted to dissect KLRG2-mediated tumor-immune crosstalk.

## 1. Introduction

Endometrial cancer (EC) has emerged as one of the most prevalent malignancies of the female reproductive system worldwide [1], exhibiting a marked upward incidence trend over recent decades, particularly in developed countries [2,3]. Although patients with early-stage disease generally achieve favorable outcomes through surgical intervention and adjuvant therapies, those with advanced disease face poor prognoses characterized by frequent recurrence and metastasis, demonstrating a five-year survival rate as low as 17–20% [4]. Notably, EC incidence exhibits a bimodal age distribution, with peak aggressiveness observed in postmenopausal women (>60 years), who account for 40–50% of cases and demonstrate higher frequencies of high-grade histology, non-endometrioid subtypes, and advanced-stage disease [1,5]. The identification of clinically actionable biomarkers for precise prognostic evaluation and personalized therapeutic strategies remains an unmet need in EC management.

Killer cell lectin-like receptor subfamily G member 2 (KLRG2), a C-type lectin-like receptor, regulates tumor progression and immune responses. In gastric cancer, KLRG2 promotes malignancy via ERK1/2 and JAK/STAT activation while suppressing p53/p38 MAPK, correlating with poor survival [6]. Elevated KLRG2 in lung adenocarcinoma marks pyroptosis-related subtypes and adverse prognosis [7]. Notably, in prostate cancer, KLRG2s association with disease aggressiveness stems primarily from a germline genetic variant (rs17160911) [8]—a mechanism distinct from somatic transcriptional dysregulation observed in other carcinomas. KLRG2 is transcriptionally induced by p53 under DNA damage [9] and contributes to genomic instability-driven models predicting chemotherapy response in lung adenocarcinoma [10]. Despite proximity to CLEC2L, KLRG2 independently modulates Th2 immunity during infections [11,12]. Given the hormone-responsive microenvironment and immune-privileged nature of the endometrium, KLRG2s roles in EC may involve unique tissue-specific mechanisms. These multifaceted roles underscore KLRG2 as a critical oncogenic effector and therapeutic target. Despite these advances, the expression patterns, clinicopathological correlations, and mechanistic implications of KLRG2 in EC remain largely unexplored.

Focusing on fundamental molecular mechanisms underlying EC progression, this study first utilizes publicly available multi-omics data to comprehensively characterize KLRG2. External validations are supplemented by independent East Asian cohorts (Nanjing, China; N = 28) and public datasets. Based on its established oncogenic and immunomodulatory roles in other malignancies, we specifically address three core questions in EC: (1) Is KLRG2 overexpression driven by promoter hypomethylation? (2) Does KLRG2 induce cytotoxic lymphocyte (CD8+/NK) depletion to shape immunosuppression? (3) Can KLRG2 serve as an independent prognostic biomarker? To mechanistically resolve these questions, we decode epigenetic regulation through methylation-transcriptome correlation analysis, quantify cytotoxic depletion via deconvolution algorithms (TIMER/ssGSEA), and establish prognostic power through multivariate Cox regression with time-dependent receiver operating characteristic (ROC). These integrated analyses position KLRG2 as a dual diagnostic-prognostic biomarker, providing rationale for targeted therapy development in multi-ethnic populations.

## 2. Materials and Methods

### 2.1. Data Collection

Transcriptomic profiles and clinical data for uterine corpus endometrial carcinoma (UCEC) and paired adjacent normal tissues were retrieved from The Cancer Genome Atlas (TCGA; https://portal.gdc.cancer.gov/) (accessed on 22 December 2024), with RNA-seq data normalized to transcripts per million (TPM) values. Pan-cancer RNA-seq data from TCGA and the Genotype-Tissue Expression (GTEx) project, uniformly processed through the TOIL pipeline to eliminate cross-study technical biases, were obtained from the University of California, Santa Cruz (UCSC) Xena platform (https://xenabrowser.net/) (accessed on 22 December 2024) [13]. For external validation of KLRG2 expression differences, we obtained mRNA data from the GSE106191 (GPL570) dataset [14] through the Gene Expression Omnibus (GEO; https://ncbi.nlm.nih.gov/geo/) (accessed on 22 December 2024). Immunohistochemical (IHC) staining results for KLRG2 in UCEC were acquired from the Human Protein Atlas (HPA; https://www.proteinatlas.org/) (accessed on 24 December 2024) [15].

### 2.2. Diagnostic Evaluation, Survival Analysis, and Nomogram Construction

For diagnostic assessment, ROC curves were generated with the “pROC” package (v1.18.0) [16] to evaluate the ability of KLRG2 expression to distinguish EC from normal tissues, with diagnostic accuracy quantified by the area under the curve (AUC). Precision-recall (PR) curves were added to evaluate class imbalance effects. Prognostic evaluation was performed using Kaplan–Meier survival curves and Cox proportional hazards models implemented in the “survival” package (v3.5.8). Variables with *p* < 0.1 in univariate Cox regression were included in multivariate analysis, and results were visualized as forest plots via “ggplot2” (v3.5.1). To predict survival probabilities, time-dependent ROC analysis for 1-, 3-, and 5-year outcomes was performed with the “timeROC” package (v0.4) [17]. Subsequently, a nomogram integrating independent prognostic factors was constructed, and its calibration was assessed using calibration curves generated by the “rms” package (v6.7.1).

### 2.3. Differential Expression and Functional Enrichment

UCEC cases were stratified into KLRG2 expression-defined subgroups (high/low stratification by median cutoff). Differentially expressed genes (DEGs) were identified using “DESeq2” (v1.42.1) [18] with thresholds of adjusted *p* < 0.05 and |log2FoldChange (FC)| > 2. Gene Ontology (GO), Kyoto Encyclopedia of Genes and Genomes (KEGG), and Gene Set Enrichment Analysis (GSEA) were performed with “ClusterProfiler” (v4.10.1) [19]. GSEA utilized the “c2.cp.all.v2022.1.Hs.symbols.gmt” gene set collection, with significance defined as adjusted *p* < 0.05 and false discovery rate (FDR) < 0.25.

### 2.4. Protein-Protein Interaction (PPI) Network

The STRING database (v12.0; confidence score > 0.5) (https://cn.string-db.org/) (accessed on 24 December 2024) [20] was used to construct a PPI network for KLRG2 and its top ten interacting partners.

### 2.5. Genetic Mutations and Methylation Analysis

KLRG2 mutations and copy number variations (CNVs) were analyzed using the cBioPortal platform (https://www.cbioportal.org/) (accessed on 24 December 2024) [21]. Promoter methylation levels in UCEC were assessed via the UALCAN web portal (https://ualcan.path.uab.edu/) (accessed on 24 December 2024) [22], and survival correlations were evaluated using the MethSurv tool (https://biit.cs.ut.ee/methsurv/) (accessed on 24 December 2024) [23].

### 2.6. Immune Microenvironment Profiling

To profile the immune landscape, immune cell infiltration scores were first quantified via single-sample gene set enrichment analysis (ssGSEA) using the “GSVA” package (v1.50.5) [24]. The abundance of six major immune cell subsets (CD8+ T cells, B cells, CD4+ T cells, macrophages, dendritic cells, and neutrophils) was further estimated using TIMER platform (https://cistrome.shinyapps.io/timer/) (accessed on 24 December 2024) [25]. Spearman correlation analysis revealed associations between KLRG2 expression and immune checkpoint markers (CTLA4, PDCD1, CD274, HAVCR2). Additionally, stromal and immune scores were derived from the “ESTIMATE” package (v1.0.13) [26], with group differences assessed by the Wilcoxon rank-sum test.

### 2.7. Quantitative Real-Time PCR (qRT-PCR) Analysis

Total RNA was isolated using the FastPure Cell/Tissue Total RNA Isolation Kit V2 (Vazyme, Nanjing, China) from 14 endometrial carcinoma tissues and 14 proliferative-phase endometrial controls. The RNA samples were reverse transcribed using HiScript III Reverse Transcriptase (Vazyme, Nanjing, China); then qRT-PCR was conducted using the ChamQ SYBR qPCR Master Mix (High ROX Premixed) on Applied Biosystems StepOnePlus. Relative expression was calculated by the 2^−ΔΔCt^ method. Primer sequences were the following: GAPDH primers: forward TGGATTTGGACGCATTGGTC, reverse TTTGCACTGGTACGTGTTGAT; KLRG2 primers: forward CTGAGGACGGCGAGGACAATC, reverse GGCTGCCCAAGCTCTCAACT.

### 2.8. Statistical Methods

All statistical analyses were conducted using R (version 4.3.3). Continuous variables were assessed for normality using the Shapiro–Wilk test (parametric tests applied when *p* ≥ 0.05), with group comparisons performed using the unpaired Student’s *t*-test or Mann–Whitney U test for independent samples and the paired t-test or Wilcoxon signed-rank test for matched samples. Categorical variables were analyzed using the χ^2^ test with Yates’ continuity correction (applied when expected cell frequencies < 5) and univariate logistic regression reporting odds ratios (OR) with 95% confidence intervals (CI). Survival analysis included Kaplan–Meier curves compared by log-rank test (stratified at median KLRG2 expression) and Cox proportional hazards regression with multivariable adjustment for predefined clinical covariates: histologic grade, myometrial invasion depth, age, pathological type, and FIGO stage; proportional hazards assumption was verified via Schoenfeld residuals (global *p* > 0.05 indicating no violation). Diagnostic modeling involved ROC curve analysis with unadjusted models (KLRG2 expression only), AUC with 95% CI computed via DeLong’s variance estimation method, and time-dependent AUC estimation using inverse probability of censoring weighting.

All computational tools and databases are comprehensively listed in Table S1 with version information and access links.

## 3. Results

### 3.1. High Expression of KLRG2 in EC

**Figure 1:**
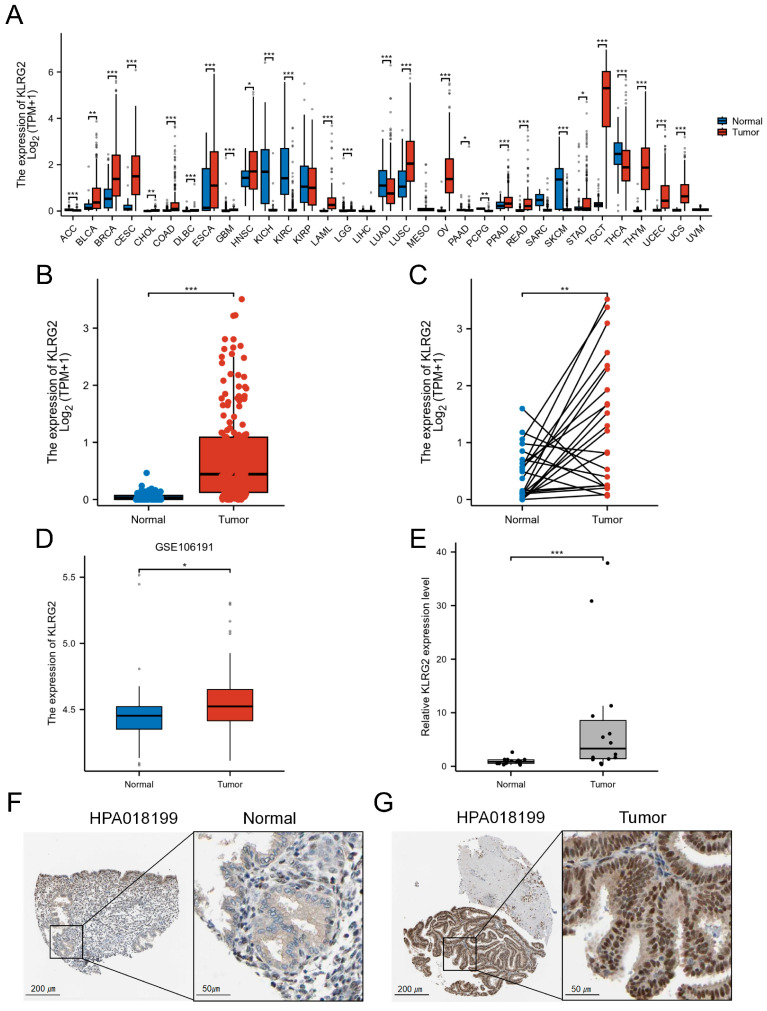
Elevated expression of KLRG2 in EC. (**A**) Pan-cancer analysis of KLRG2 in TCGA and GTEx databases. (**B**) Unpaired analysis between EC and normal endometrium in TCGA database. (**C**) Paired analysis between EC and adjacent normal endometrium in TCGA database. (**D**) Validation of KLRG2 overexpression in EC using GSE106191 dataset. (**E**) qPCR validation of KLRG2 overexpression in clinical samples (EC and proliferative-phase endometrium). (**F**,**G**) IHC validation of KLRG2 expression in normal endometrium (**F**) and EC (**G**) from HPA. (* *p* < 0.05; ** *p* < 0.01; *** *p* < 0.001).

Pan-cancer analysis utilizing TCGA and GTEx databases revealed significant overexpression of KLRG2 across multiple malignancies, including EC, ovarian cancer, cervical cancer, bladder cancer, breast cancer, esophageal carcinoma, testicular cancer, and lung adenocarcinoma (Figure 1A). Compared with normal endometrial tissues, KLRG2 expression was markedly upregulated in EC specimens (W = 1895, *p* < 0.001) (Figure 1B). Paired analysis of 23 EC tissues and adjacent normal counterparts demonstrated significantly higher KLRG2 levels in tumor tissues (t(22) = 3.414, *p* = 0.0025) (Figure 1C). Validation in the GSE106191 (GPL570) dataset confirmed KLRG2 overexpression in endometrial carcinoma (W = 752.5, *p* = 0.021) (Figure 1D). To further strengthen this finding, qPCR analysis of our independently collected clinical samples (14 EC tissues and 14 proliferative-phase endometrial controls) showed a striking elevation of KLRG2 expression in EC compared to normal endometrium (W = 29, *p* = 0.001) (Figure 1E). IHC data from HPA revealed stronger KLRG2 staining intensity in EC tissues compared to normal endometrium (Figure 1G–F).

### 3.2. Association of KLRG2 Expression with Adverse Clinicopathological Features and Prognosis

**Figure 2:**
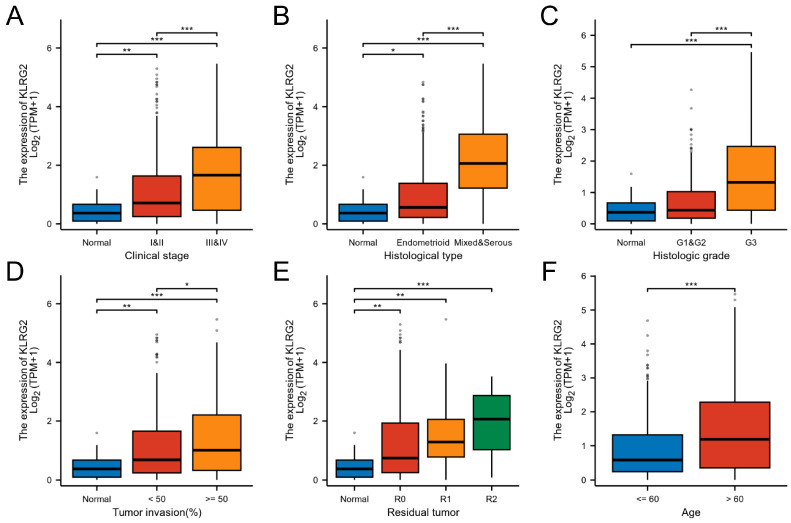
Expression of KLRG2 in different clinicopathological characteristics. (**A**) Clinical stage; (**B**) Histological type; (**C**) Histologic grade; (**D**) Tumor invasion; (**E**) Residual tumor; (**F**) Age. (* *p* < 0.05; ** *p* < 0.01; *** *p* < 0.001).

KLRG2 expression demonstrated significant variations across FIGO stages, histologic subtypes, tumor grades, residual tumor status, and myometrial invasion depth (*p* < 0.05) (Table 1; Figure 2A–F). Univariate logistic regression identified strong correlations between KLRG2 expression and key clinicopathological parameters, particularly histologic subtype (OR = 7.823, 95% CI = 14.833–12.664, *p* < 0.001), tumor grade (OR = 3.713, 95% CI = 2.579–5.346, *p* < 0.001), residual tumor status (OR = 3.680, 95% CI = 1.696–7.985, *p* < 0.001), FIGO stage (OR = 2.426, 95% CI = 1.655–3.557, *p* < 0.001), and age (OR = 2.197, 95% CI = 1.544–3.126, *p* < 0.001) (Table 2).

**Figure 3:**
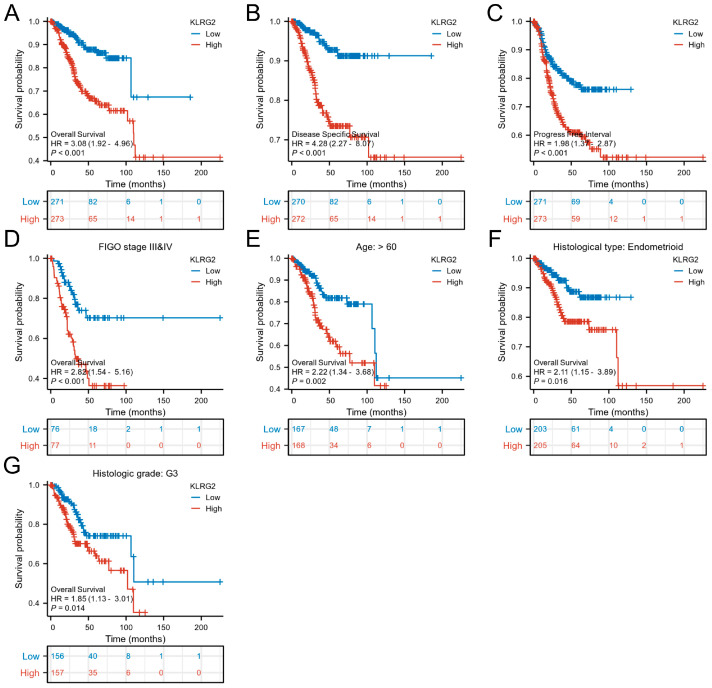
Kaplan–Meier survival analyses of KLRG2 expression. (**A**–**C**) Survival curves for (**A**) OS (HR = 3.08, 95% CI = 1.92–4.96), (**B**) DSS (HR = 4.28, 95% CI = 2.27–8.07), and (**C**) PFI (HR = 1.98, 95% CI = 1.37–2.87) stratified by median KLRG2 expression. (**D**–**G**) Subgroup analyses in (**D**) FIGO III/IV disease (HR = 2.82, 95% CI = 1.54–5.16), (**E**) patients aged > 60 years (HR = 2.22, 95% CI = 1.34–3.68), (**F**) endometrioid carcinoma (HR = 2.11, 95% CI = 1.15–3.89), and (**G**) G3 tumors (HR = 1.85, 95% CI = 1.13–3.01).

Kaplan–Meier analysis stratified by median KLRG2 expression revealed that high-KLRG2 patients exhibited worse overall survival (OS: HR = 3.08, 95% CI = 1.92–4.96, *p* < 0.001), disease-specific survival (DSS: HR = 4.28, 95% CI = 2.27–8.07, *p* < 0.001), and progression-free interval (PFI: HR = 1.98, 95% CI = 1.37–2.87, *p* < 0.001) (Figure 3A–C). Subgroup analysis confirmed the prognostic significance of KLRG2 high expression in patients aged > 60 years, G3 tumors, endometrioid carcinoma, and FIGO III and IV disease (Figure 3D–G).

### 3.3. Clinical Significance of KLRG2 in EC

**Figure 4:**
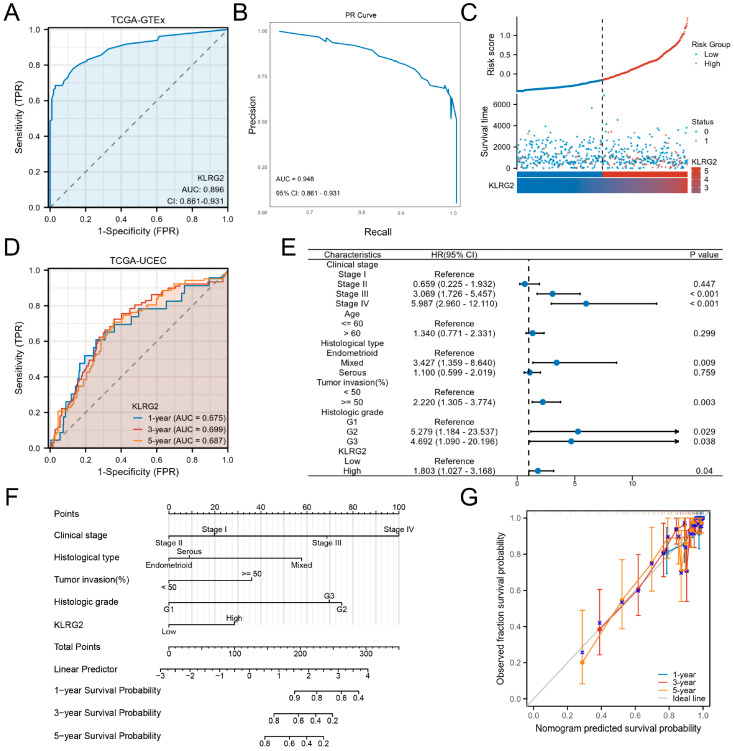
Clinical Significance of KLRG2 in EC. (**A**) ROC curve and (**B**) PR curve analysis of KLRG2 for distinguishing EC from normal tissues in TCGA and GTEx. (**C**) Risk stratification landscape based on KLRG2 expression profiles in TCGA-UCEC cohort. (**D**) Time-dependent ROC analysis of KLRG2s predictive capacity for survival in the TCGA-UCEC cohort. (**E**) Forest plots of hazard ratios from multivariate Cox regression analysis for OS. (**F**) Nomogram integrating KLRG2 with clinical parameters. (**G**) Calibration plots of the nomogram.

ROC analysis demonstrated strong diagnostic performance for KLRG2 (AUC = 0.896) in distinguishing EC (Figure 4A). To address potential class imbalance limitations of ROC metrics, PR curve analysis was performed, yielding an AUC-PR of 0.948 (95% CI: 0.861–0.931) (Figure 4B), confirming robust predictive accuracy. Risk stratification based on KLRG2 expression revealed shorter time-to-event in high-risk groups (Figure 4B). Time-dependent ROC curves confirmed prognostic utility for 1-, 3-, and 5-year survival (Figure 4C).

Multivariate Cox regression identified high KLRG2 expression, G2/G3 grade, deep myometrial invasion, and advanced FIGO stage as independent predictors of OS (Figure 4D). A prognostic nomogram integrating these factors effectively predicted 1-, 3-, and 5-year survival rates (Figure 4E). Calibration plots demonstrated excellent model performance (Figure 4F).

### 3.4. DEGs and Functional Enrichment Analysis

**Figure 5:**
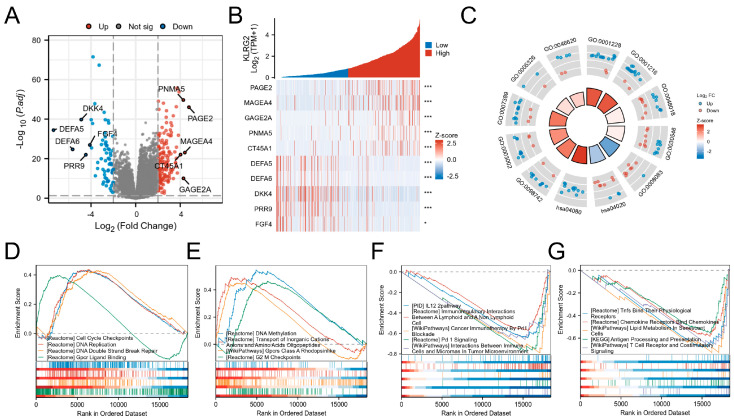
Analysis of KLRG2-related differentially expressed genes (DEGs) in EC. (**A**) Volcano plot of DEGs between KLRG2 high- and low-expression groups. (**B**) Heatmap of the top 5 upregulated and downregulated genes associated with KLRG2 expression. (**C**) Circular diagrams of GO and KEGG enrichment analyses for DEGs. (**D**,**E**) GSEA analyses of the KLRG2 high-expression group. (**F**,**G**) GSEA analyses of the KLRG2 low-expression group. (* *p* < 0.05; *** *p* < 0.001).

Comparative transcriptomics identified 223 DEGs (157 upregulated and 66 downregulated; adjusted *p* < 0.05 and |log2FC| > 2) between KLRG2-high and -low groups (Figure 5A). Top DEGs included PAGE2, MAGEA4, GAGE2A, PNMA5, CT45A1 (upregulated), and DEFA5, DEFA6, DKK4, PRR9, and FGF4 (downregulated) (Figure 5B).

GO analysis revealed enrichment in cell adhesion, regionalization, and growth regulation processes (GO:0003002: regionalization; GO:0007389: pattern specification process; GO:0046620: regulation of organ growth; GO:0098742: cell-cell adhesion via plasma-membrane adhesion molecules). Molecular functions included transcription activator activity and receptor signaling (GO:0001228: DNA-binding transcription activator activity, RNA polymerase II-specific; GO:0048018: receptor ligand activity; GO:0030546: signaling receptor activator activity; GO:0008083: growth factor activity; GO:0005326: neurotransmitter transmembrane transporter activity). KEGG pathways involved calcium signaling (hsa04020) and neuroactive ligand-receptor interactions (hsa04080) (Figure 5C). GSEA highlighted proliferation-related pathways (cell cycle checkpoints, DNA repair) in KLRG2-high tumors versus immune microenvironment regulation in KLRG2-low groups (Figure 5D–G).

### 3.5. PPI Network and Functional Partners of KLRG2

**Figure 6:**
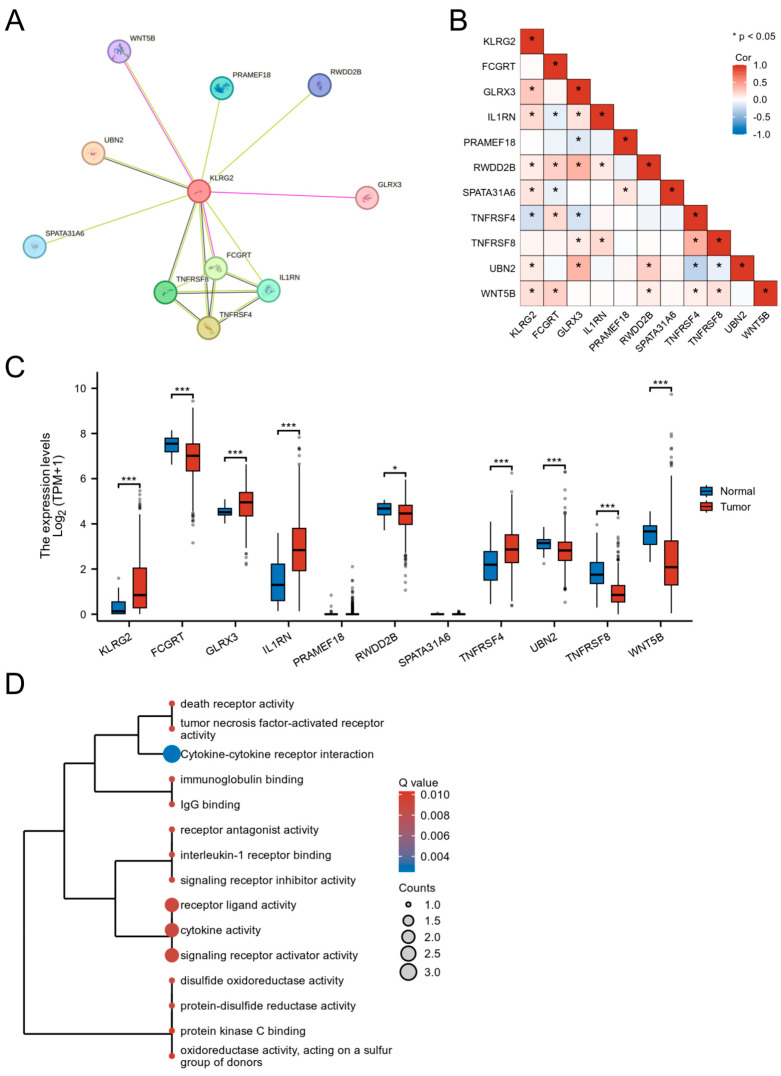
Functional landscape of KLRG2-interacting genes in EC. (**A**) STRING-based PPI network identifying 10 potential interactors. (**B**) Co-expression heatmap between KLRG2 and its interacting partners. (**C**) Tumor-specific dysregulation of KLRG2 interactors. (**D**) Functional enrichment analysis of KLRG2-interacting genes through GO and KEGG. (* *p* < 0.05; *** *p* < 0.001).

STRING-based PPI network analysis predicted 10 potential interactors of KLRG2, including FCGRT, GLRX3, and WNT5B (Figure 6A). Transcriptomic correlation analysis in TCGA-UCEC validated significant correlations between KLRG2 and seven candidates (GLRX3, IL1RN, RWDD2B, SPATA31A6, TNFRSF4, UBN2, and WNT5B) (Figure 6B). Comparative analysis revealed tumor-specific upregulation of GLRX3, IL1RN, and TNFRSF4 (*p* < 0.001), contrasting with downregulation of FCGRT, UBN2, and WNT5B in EC versus normal tissues (Figure 6C). Functional enrichment highlighted predominant involvement in cytokine-mediated pathways (e.g., TNF receptor superfamily) and redox regulation (Figure 6D).

### 3.6. Genetic Mutation and Methylation of KLRG2

**Figure 7:**
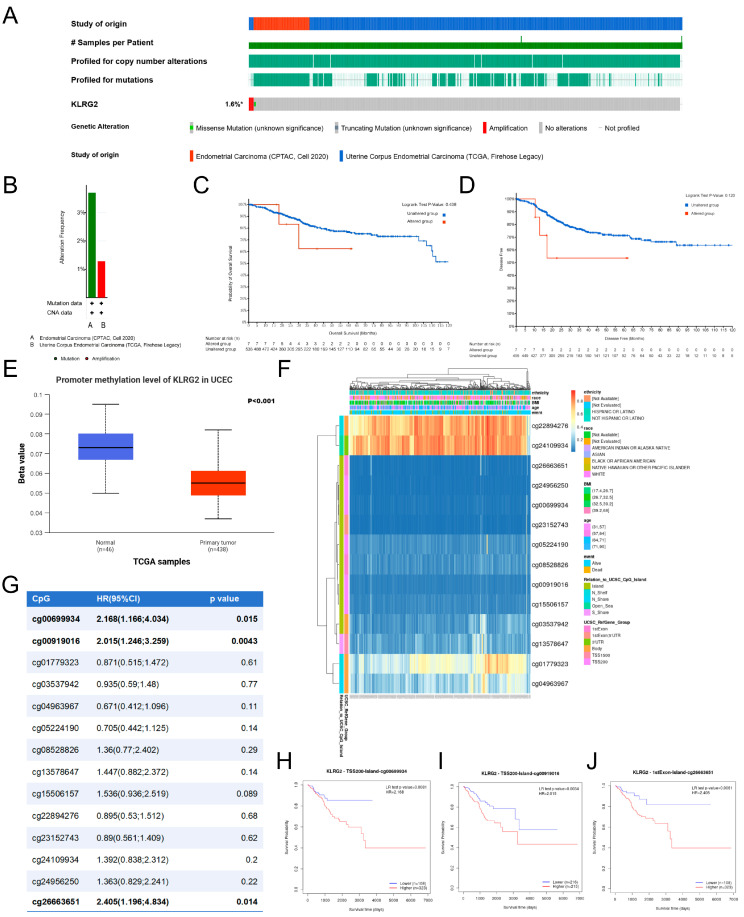
Analysis of KLRG2 somatic mutations and DNA methylation. (**A**,**B**) Mutation profiles visualized via cBioPortal OncoPrint platform. (**C**,**D**) Survival analysis comparing OS (**C**) and PFS (**D**) outcomes between mutation carriers and wild-type cases. (**E**) KLRG2 methylation levels in EC. (**F**) Methylation-transcription correlation map identifying epigenetic silencing patterns (MethSurv). (**G**) Association between CpG site methylation and OS. (**H**–**J**) Kaplan–Meier analysis of OS for KLRG2 methylation at CpG sites (**H**) cg00699934, (**I**) cg00919016, and (**J**) cg26663651. (* not all samples are profiled).

Analysis of two EC cohorts from cBioPortal (CPTAC 2020, n = 81; TCGA Firehose Legacy, n = 549) revealed KLRG2 genetic alterations (1.6% frequency), including missense mutations and copy-number amplifications (Figure 7A,B). Kaplan–Meier analysis showed no survival difference between alteration carriers and wild-type cases (OS *p* = 0.438; DFS *p* = 0.120) (Figure 7C,D).

As shown in Figure 7E, DNA methylation analysis via UALCAN demonstrated decreased promoter methylation of KLRG2 in EC compared to normal endometrium (*p* < 0.001). MethSurv database analysis identified three hypermethylated CpG sites (cg00699934, cg00919016, cg26663651) associated with poor prognosis, despite predominant hypomethylation across the gene region (Figure 7F–J).

### 3.7. KLRG2 Correlation with Immune Infiltration and Checkpoints

**Figure 8:**
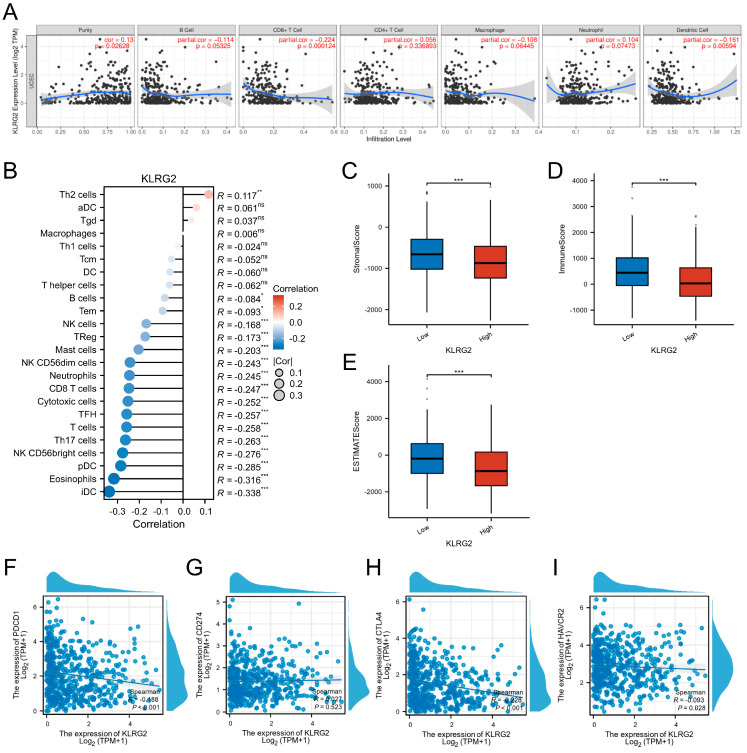
KLRG2-immune microenvironment and checkpoint correlations in EC. (**A**) KLRG2 correlation with tumor purity and immune cell infiltration. (**B**) Lollipop plot mapping KLRG2 expression correlations with 24 immune cell subtypes. (**C**–**E**) Comparative TME scoring ((**C**) stromal, (**D**) immune, (**E**) ESTIMATE) across KLRG2-high and -low expression groups. (**F**–**I**) Scatter plots of correlation analysis between KLRG2 and (**F**) PDCD1, (**G**) CD274, (**H**) CTLA4, and (**I**) HAVCR2. (*** *p* < 0.001; ** *p* < 0.01; * *p* < 0.05; ns: not significant).

Analysis of the TIMER database revealed significant associations between KLRG2 expression and tumor immune microenvironment parameters in EC. The expression of KLRG2 was positively correlated with tumor purity (Spearman’s ρ = 0.13, *p* = 0.026), while demonstrating negative correlations with infiltration levels of dendritic cells (Spearman’s ρ = −0.161, *p* < 0.001) and CD8+ T cells (Spearman’s ρ = −0.224, *p* < 0.001), as shown in Figure 8A.

Further evaluation using the ssGSEA algorithm demonstrated a positive association between KLRG2 expression and Th2 cell abundance (Spearman’s ρ = 0.117, *p* = 0.006). Conversely, significant negative correlations were observed with both cytotoxic and immunoregulatory immune subsets: Cytotoxic populations included NK CD56bright cells (Spearman’s ρ = −0.276, *p* < 0.001) and CD8+ T cells (Spearman’s ρ = −0.247, *p* < 0.001), while immunomodulatory components comprised immature dendritic cells (iDC; Spearman’s ρ = −0.338, *p* < 0.001), plasmacytoid dendritic cells (pDC; Spearman’s ρ = −0.285, *p* < 0.001), eosinophils (Spearman’s ρ = −0.316, *p* < 0.001), and Th17 cells (Spearman’s ρ = −0.263, *p* < 0.001) (Figure 8B). ESTIMATE algorithm-based scoring revealed significantly lower stromal, immune, and composite ESTIMATE scores in the KLRG2-high group compared to the low-expression cohort (*p* < 0.001) (Figure 8C–E).

In-depth analysis of immune checkpoint molecules using TCGA-UCEC data identified significant negative correlations between KLRG2 expression and PD-1 (PDCD1; Spearman’s ρ = −0.188, *p* < 0.001) as well as CTLA-4 (Spearman’s ρ = −0.228, *p* < 0.001) (Figure 8F–I).

## 4. Discussion

This integrative multi-omics study establishes KLRG2 as a pivotal orchestrator of endometrial carcinogenesis, coordinating tumor-intrinsic proliferation, epigenetic reprogramming, and microenvironmental immunosuppression. Our findings position KLRG2 as a dual diagnostic-prognostic biomarker and immunomodulatory target with high translational relevance.

Oncogenic driver functions are evidenced by KLRG2s pan-cancer overexpression, with marked EC-specific upregulation correlating with aggressive clinicopathological features, including advanced FIGO stage, deep myometrial invasion, and high-grade histology (particularly non-endometrioid subtypes) [4]. Critically, high KLRG2 expression independently predicts adverse survival outcomes across multiple endpoints, with pronounced effects in high-risk subgroups. Mechanistically, functional enrichment directly links KLRG2 to core proliferation pathways (G2/M checkpoint, DNA replication/repair) and GPCR signaling, providing a molecular basis for its role in genomic instability and therapeutic resistance [27,28,29].

The diagnostic and prognostic performance of KLRG2, highlighted by an AUC of 0.896 in ROC analysis and the predictive accuracy of the nomogram, positions it as a viable biomarker for early detection and risk stratification. The integration of KLRG2 into existing prognostic models could refine patient stratification, particularly for identifying candidates likely to benefit from adjuvant therapy or immune modulation.

Epigenetic dysregulation plays a pivotal role in KLRG2 overexpression, with promoter hypomethylation identified as a key driver of its transcriptional activation [30,31]. Paradoxically, hypermethylation at specific intragenic CpG sites (e.g., cg00919016) is associated with poorer prognosis. This dual methylation pattern mirrors observations in SUMF2 [32], where tumor-specific CpG_3 and CpG_7 hypermethylation correlates with shortened progression-free survival, potentially through transcript stabilization or alternative splicing mechanisms—processes also implicated in LINE-1 hypomethylation-related oncogenesis [33,34]. This epigenetic duality underscores the complexity of KLRG2 regulation, where baseline overexpression may be amplified by post-transcriptional modifications. The lack of prognostic significance for somatic KLRG2 mutations contrasts with its epigenetic regulation, underscoring the primacy of expression-level dysregulation in EC progression.

KLRG2 expression is closely associated with the immune landscape of EC. Its positive correlation with tumor purity and negative associations with CD8+ T cell and DC infiltration suggest its role in promoting an immune-excluded TME, a phenotype linked to poor prognosis and treatment resistance [35,36]. Furthermore, the contrasting positive correlation with Th2 cell abundance and negative correlations with cytotoxic (NK CD56bright cells, CD8+ T cells) and immunoregulatory subsets (iDCs, pDCs, eosinophils, Th17 cells) highlight KLRG2s potential to drive a Th2-skewed, immunosuppressive TME [37,38]. This Th2 dominance may promote M2 macrophage polarization and angiogenesis while suppressing Th1-mediated anti-tumor immunity, fostering tumor progression [39,40]. Consistently, lower stromal, immune, and composite ESTIMATE scores in KLRG2-high tumors further support its role in shaping an immunosuppressive TME [41]. This immune-excluded phenotype presents a key therapeutic challenge consistent with immunotherapy-resistant EC subtypes [42]. The inverse correlation with checkpoint molecules (PD-1/CTLA-4) likely reflects restricted lymphocyte infiltration rather than altered exhaustion states, a distinction critical for biomarker-guided combination strategies [43].

While this study provides a comprehensive multi-omics characterization of KLRG2, three key limitations should be noted. First, the retrospective design and reliance on bulk transcriptomic data may obscure cell-type-specific interactions, particularly within the TME. Second, the proposed mechanisms linking KLRG2 to immune evasion remain hypothetical and require validation through functional studies such as genetic knockout models or co-culture assays. Finally, the paradoxical methylation patterns observed in KLRG2 necessitate locus-specific epigenetic analyses to resolve their functional significance. To bridge these gaps, we propose three translational research priorities: (i) Spatial multi-omics (single-cell RNA-seq/imaging mass cytometry) to resolve KLRG2s cell type-specific immunosuppression; (ii) CRISPR-based functional validation of methylation-regulated transcript stability; (iii) therapeutic profiling of KLRG2-blocking antibodies in patient-derived organoids combined with anti-PD-1/CTLA-4.

## 5. Conclusions

In summary, KLRG2 emerges as a linchpin of endometrial cancer progression, integrating proliferative signaling, epigenetic reprogramming, and immune microenvironment remodeling. Its dual role as a prognostic biomarker and immune modulator highlights its clinical relevance for personalized therapy. The antagonism between KLRG2-driven oncogenesis and immune suppression underscores the need for combination therapies targeting both tumor-intrinsic pathways and extrinsic immune escape mechanisms. Translational validation of KLRG2 as a therapeutic target and biomarker holds promise for improving outcomes in high-risk EC patients, bridging the gap between molecular insights and clinical application.

## Figures and Tables

**Table 1:** Association of KLRG2 expression with clinicopathological characteristics in the TCGA-UCEC cohort.

Characteristics	Low Expression of KLRG2	High Expression of KLRG2	*p*-Value ^1^
n	277	277	
Age, n (%)			<0.001
<=60	128 (61.8%)	79 (38.2%)	
>60	146 (42.4%)	198 (57.6%)	
Race, n (%)			0.070
Asian	10 (50%)	10 (50%)	
Black or African American	44 (40.4%)	65 (59.6%)	
White	201 (52.9%)	179 (47.1%)	
BMI, n (%)			0.054
<=30	95 (44.8%)	117 (55.2%)	
>30	165 (53.4%)	144 (46.6%)	
Menopause status, n (%)			0.034
Pre	24 (68.6%)	11 (31.4%)	
Peri	11 (64.7%)	6 (35.3%)	
Post	220 (48.4%)	235 (51.6%)	
Diabetes, n (%)			0.585
No	166 (50.5%)	163 (49.5%)	
Yes	59 (47.6%)	65 (52.4%)	
Clinical stage, n (%)			<0.001
Stage I	199 (58%)	144 (42%)	
Stage II	23 (44.2%)	29 (55.8%)	
Stage III	50 (38.5%)	80 (61.5%)	
Stage IV	5 (17.2%)	24 (82.8%)	
Histological type, n (%)			<0.001
Endometrioid	253 (61.4%)	159 (38.6%)	
Serous	13 (11%)	105 (89%)	
Mixed	11 (45.8%)	13 (54.2%)	
Histologic grade, n (%)			<0.001
G1	75 (75.8%)	24 (24.2%)	
G2	78 (64.5%)	43 (35.5%)	
G3	123 (38.1%)	200 (61.9%)	
Tumor invasion (%), n (%)			0.021
<50	149 (57.1%)	112 (42.9%)	
>=50	100 (46.5%)	115 (53.5%)	
Residual tumor, n (%)			0.002
R0	201 (53.3%)	176 (46.7%)	
R1	6 (27.3%)	16 (72.7%)	
R2	3 (18.8%)	13 (81.2%)	
Primary therapy outcome, n (%)			0.041 ^2^
PD	6 (30%)	14 (70%)	
SD	3 (50%)	3 (50%)	
PR	3 (25%)	9 (75%)	
CR	241 (54.3%)	203 (45.7%)	
Hormones therapy, n (%)			0.521
No	155 (51.8%)	144 (48.2%)	
Yes	22 (46.8%)	25 (53.2%)	
Radiation therapy, n (%)			0.367
No	147 (52.3%)	134 (47.7%)	
Yes	120 (48.4%)	128 (51.6%)	
OS event, n (%)			<0.001
Alive	253 (55%)	207 (45%)	
Dead	24 (25.5%)	70 (74.5%)	
DSS event, n (%)			<0.001
No	264 (54%)	225 (46%)	
Yes	12 (19%)	51 (81%)	
PFI event, n (%)			<0.001
No	234 (55.1%)	191 (44.9%)	
Yes	43 (33.3%)	86 (66.7%)	

PD, Progressive disease; SD, Stable disease; PR, Partial response; CR, Complete response; OS, Overall survival; DSS, Disease-specific survival; PFI, Progression-free interval. ^1^ Chi-square test. ^2^ Yates’ continuity correction.

**Table 2:** Logistic regression analysis of KLRG2 expression and clinicopathological parameters.

Characteristics	Total (N)	OR (95% CI)	*p*-Value
Clinical stage (Stage III and Stage IV vs. Stage I and Stage II)	554	2.426 (1.655–3.557)	<0.001
Age (>60 vs. <=60)	551	2.197 (1.544–3.126)	<0.001
BMI (>30 vs. <=30)	521	0.709 (0.499–1.007)	0.054
Histological type (Mixed and Serous vs. Endometrioid)	554	7.823 (4.833–12.664)	<0.001
Tumor invasion(%) (>=50 vs. <50)	476	1.530 (1.064–2.200)	0.022
Diabetes (Yes vs. No)	453	1.122 (0.742–1.696)	0.585
Residual tumor (R1 and R2 vs. R0)	415	3.680 (1.696–7.985)	<0.001
Menopause status (Post vs. Pre and Peri)	507	2.199 (1.197–4.039)	0.011
Histologic grade (G3 vs. G1 and G2)	543	3.713 (2.579–5.346)	<0.001

OR, Odds Ratio; CI, Confidence Interval.

## Data Availability

The experimental data generated in this study are available from the corresponding author upon reasonable request. The remaining raw data were obtained from multiple public databases, including TCGA (https://portal.gdc.cancer.gov/, accessed on 22 December 2024), GEO (https://www.ncbi.nlm.nih.gov/geo/, accessed on 22 December 2024), UCSC XENA (https://xenabrowser.net/, accessed on 22 December 2024), cBioPortal (https://www.cbioportal.org/, accessed on 24 December 2024), HPA (https://www.proteinatlas.org/, accessed on 24 December 2024), STRING database (version 12.0, https://cn.string-db.org/, accessed on 24 December 2024), UALCAN (https://ualcan.path.uab.edu/, accessed on 24 December 2024), and MethSurv (https://biit.cs.ut.ee/methsurv/, accessed on 24 December 2024). Custom R scripts used for bioinformatics analyses are available from the corresponding author upon reasonable request for academic non-commercial use.

## Supplementary Materials

The following supporting information can be downloaded at: https://www.mdpi.com/article/10.3390/biomedicines13071592/s1.

## Abbreviations

The following abbreviations are used in this manuscript:

EC	Endometrial cancer
KLRG2	Killer cell lectin-like receptor subfamily G member 2
TME	Tumor microenvironment
UCEC	Uterine corpus endometrial carcinoma
TPM	Transcripts per million
GTEx	Genotype-Tissue Expression
GEO	Gene Expression Omnibus
IHC	Immunohistochemical
HPA	Human Protein Atlas
ROC	Receiver operating characteristic
AUC	Area under the curve
DEGs	Differentially expressed genes
GO	Gene Ontology
KEGG	Kyoto Encyclopedia of Genes and Genomes
GSEA	Gene Set Enrichment Analysis
FDR	False discovery rate
PPI	Protein-Protein Interaction
CNVs	Copy number variations
ssGSEA	Single-sample gene set enrichment analysis
qRT-PCR	Quantitative Real-Time PCR
OS	Overall survival
DSS	Disease-specific survival
PFI	Progression-free interval
